# Immunotherapy for Pediatric Acute Lymphoblastic Leukemia: Recent Advances and Future Perspectives

**DOI:** 10.3389/fimmu.2022.921894

**Published:** 2022-06-13

**Authors:** Meng Lv, Yan Liu, Wei Liu, Yabing Xing, Shengnan Zhang

**Affiliations:** ^1^ Department of Pharmacy, Children’s Hospital Affiliated to Zhengzhou University, Zhengzhou, China; ^2^ Department of Pharmacy, The First Affiliated Hospital of Zhengzhou University, Zhengzhou, China; ^3^ Department of Hematology Oncology, Children’s Hospital Affiliated to Zhengzhou University, Zhengzhou, China

**Keywords:** pediatric ALL, T-cell engagers, CAR T cell therapy, macrophage-based immunotherapy, NK cell-based immunotherapy

## Abstract

Pediatric acute lymphoblastic leukemia (ALL) is the most common subtype of childhood leukemia, which is characterized by the abnormal proliferation and accumulation of immature lymphoid cell in the bone marrow. Although the long-term survival rate for pediatric ALL has made significant progress over years with the development of contemporary therapeutic regimens, patients are still suffered from relapse, leading to an unsatisfactory outcome. Since the immune system played an important role in the progression and relapse of ALL, immunotherapy including bispecific T-cell engagers and chimeric antigen receptor T cells has been demonstrated to be capable of enhancing the immune response in pediatric patients with refractory or relapsed B-cell ALL, and improving the cure rate of the disease and patients’ quality of life, thus receiving the authorization for market. Nevertheless, the resistance and toxicities associated with the current immunotherapy remains a huge challenge. Novel therapeutic options to overcome the above disadvantages should be further explored. In this review, we will thoroughly discuss the emerging immunotherapeutics for the treatment of pediatric ALL, as well as side-effects and new development.

## Introduction

Acute lymphoblastic leukemia (ALL), characterized by the abnormal clonal proliferation of the early lymphoid stem cells or progenitor cells and the depletion of the normal hematopoietic cells in the marrow, is the most prevalent subtype of leukemia with a rapidly growing incidence worldwide (1–5). Although ALL occurs in both adults and children, children represent up to 80% of cases (6). Currently, improved long-term survival rates have increased to more than 90% in pediatric ALL thanks to the contemporary therapeutic regimens (7, 8). However, approximately 20% of the patients remain refractory to primary therapy or suffer from relapse after initial complete remission (CR), leading to a poor prognosis (9, 10). Therefore, the exploration of novel therapeutic approaches for pediatric refractory/relapse (R/R) ALL are urgently needed and will eventually benefit this population.

Until now, accumulating evidence has suggested that tumor microenvironment (TME) contributes to the cancer development and progression (11–13). As key members in TME, immune cells consisting of T cells, macrophages, and natural killer cells (NKs) have reduced activity and poise in an immunosuppressive state. Activating the immune system to recognize and eradicate cancer cells rather than remove or directly attack the cancer cells, termed immunotherapy, has been proposed as an alternative to conventional cancer treatment and widely explored over the last decade (14, 15). In particular, immunotherapy promoted rapid development for the treatment of the hematologic malignancies (16–18). The approval for market of blinatumomab, a bispecific CD3/CD19 T-cell engager, and tisagenlecleucel, a CAR T cell therapy, has demonstrated dramatic progress in the treatment of pediatric R/R B-cell precursor ALL (R/R BCP ALL). Combination or replacement of conventional chemotherapy with immunotherapy to further improve the cure rates and life quality of pediatric patients with ALL has become the priority issue for the moment (19–21). Generally, pediatric cancers are not smaller versions of adult cancers (6, 22). The progress and development in pediatric cancers lags behind adult patients (23). Herein, we aim to provide a comprehensive overview of the emerging immunotherapeutic approaches in pediatric ALL, thus guiding the development of novel therapeutic options. The preclinical research and ongoing clinical trials in this field will be extensively summarized in this review (**Table 1**). Due to T-cell ALL accounts for merely 15% of the pediatric ALL patients and has a different immunophenotype from B-cell ALL, immunotherapy for pediatric T-cell ALL is outside the scope of this article.

**Table 1:** Emerging immunotherapeutic approaches for pediatric B-cell ALL.

Interventions	Target	Patients number	Patients group	Indications	Study phase	Clinical Trial number	Ref.
**T cell engagers**							
Blinatumomab	CD3/CD19	93	Up to 17 years	R/R BCP ALL	Phase I/II	NCT01471782	(24, 25)
Blinatumomab	CD3/CD19	110	28 days to 18 years	R/R BCP ALL	Expanded access study	NCT02187354	(26, 27)
Blinatumomab	CD3/CD19	111	Up to 17 years	High-risk first relapse BCP ALL	Phase III	NCT02393859	(28)
Blinatumomab	CD3/CD19	670	1 to 30 years	Relapsed B-cell ALL	Phase III	NCT02101853	(29)
Blinatumomab	CD3/CD19	23	1 to 70 years	Maintenance for patients with B-cell ALL after alloHSCT	Phase II	NCT02807883	(30)
Blinatumomab	CD3/CD19	5000	Up to 18 years	ALL	Phase III	NCT03643276	(31)
CMG1A46	CD3/CD19/CD20	165	18 years and older	B-cell NHL and/or ALL	Phase I/II	NCT05348889	/
**CAR T-cell therapy (corresponding costimulatory domain)**							
Tisagenlecleucel (4-1BB)	CD19	30	5 to 20 years	R/R CD19 positive B-cell ALL	Phase I/IIa	NCT01626495	(32)
Tisagenlecleucel (4-1BB)	CD19	75	Up to 25 years	R/R B-cell ALL	Phase II	NCT02435849	(33)
Brexucabtagene autoleucel (CD28)	CD19	125	18 years and older	R/R BCP ALL	Phase I/II	NCT02614066	(34–36)
Brexucabtagene autoleucel (CD28)	CD19	116	Up to 21 years	R/R BCP ALL and R/R B-cell NHL	Phase I/II	NCT02625480	/
CD19CAR T cells (4-1BB)	CD19	167	1 to 26 years old	R/R CD19 positive leukemia	Phase I/II	NCT02028455	/
CD19CAR T cells (4-1BB)	CD19	35	Up to 21 years	R/R CD19 positive ALL	Phase I/II	NCT03573700	/
CD19CAR T cells (CD28 with or without 4-1BB)	CD19	64	Up to 75 years	Advanced B-cell NHL, ALL, and CLL	Phase I	NCT01853631	/
CD19CAR T cells (4-1BB)	CD19	27	Up to 29 years	B-cell ALL	Phase II	NCT04276870	/
CD19CAR T cells (4-1BB)	CD19	121	Up to 25 years	R/R B-cell ALL and B-cell NHL	Phase I/II	NCT03743246	/
CD19CAR T cells (CD28 or 4-1BB)	CD19	50	3 years and older	B-cell malignancy	Phase I/II	NCT02782351	/
CD19CAR T cells (CD28)	CD19	23	Up to 26 Years	Relapsed B-cell ALL	Phase I	NCT01860937	/
CD19CAR T cells (not reported)	CD19	54	3 to 70 years	R/R ALL	Phase I/II	NCT03016377	/
CD19CAR T cells (CD28)	CD19	53	1 to 30 years	B-cell leukemia or lymphoma	Phase I	NCT01593696	(37, 38)
CD22CAR T cells(4-1BB)	CD22	208	3 to 39 years	R/R CD22 positive B-cell malignancies	Phase I	NCT02315612	(39)
CD22-CAR T cells (4-1BB)	CD22	5	18 years and older	R/R B-cell ALL	Phase I	NCT02588456	(40)
CD22-CAR T cells (4-1BB)	CD22	15	1 to 24 years	R/R B-cell ALL	Phase I	NCT02650414	/
CD22CAR T cells (4-1BB)	CD22	34	1 to 55 years	R/R B-cell ALL	Observational study	ChiCTR-OIC-17013523	(41)
AUTO3 (OX40 and 4-1BB)	CD19/CD22	23	1 to 24 years	R/R B-cell ALL	Phase I/II	NCT03289455	(42)
CD19 and CD22 bispecific CAR T cells (4-1BB)	CD19/CD22	87	3 to 39 years	Recurrent or refractory CD19/CD22 positive B-cell malignancies	Phase I	NCT03448393	/
CTA101 (4-1BB)	CD19	72	3 to 70 years	R/R CD19 positive B-cell ALL and NHL	Early Phase I	NCT04227015	/
CD19CAR T cells and CD22CAR T cells (4-1BB)	CD19 and CD22	20	1 to 16 years	R/R B-cell ALL	Phase I	ChiCTR-OIB-17013670	(43)
CD19 CAR T cells (4-1BB)	CD19	32	Up to 24 Years	high risk, relapsed CD19 positive ALL and Burkitt Lymphoma	Phase I	NCT02443831	(44)
CD19 CAR T cells (4-1BB)	CD19	20	1 to 70 years	20	Phase I	ChiCTR1900024456	
**Combination therapy**							
Pembrolizumab	PD-1	12	Adults	MRD positive ALL	Phase II	NCT02767934	(107)
Blinatumomab with pembrolizumab	PD-1, CD3/CD19	24	18 years and older	R/R B-cell ALL	Phase I/II	NCT03160079	/
Blinatumomab with pembrolizumab	PD-1, CD3/CD19	36	18 years and older	recurrent or refractory ALL	Phase I/II	NCT03512405	/
Blinatumomab with nivolumab	PD-1, CD3/CD19	550	1 to 30 years	first relapsed B-cell ALL	Phase II	NCT04546399	/
Blinatumomab with chemotherapy	CD3/CD19	6720	1 to 31 years	Newly diagnosed B-cell lymphoblastic leukemia	Phase III	NCT03914625	/
**Other emerging therapeutic approaches**							
TTTI-621	CD47	260	18 years and older	Hematologic malignancies and solid tumors	Phase I	NCT02663518	/	
CAR NK cells	CD19	14	Up to 18 years	B-cell ALL	Phase I	NCT00995137	/	
CAR NK cells	CD19	20	Up to 80 years	B-cell ALL	Phase I	NCT01974479	/	
TAA-T	Tumor neoantigens	90	6 months to 80 Years	R/R hematopoietic malignancies, AML and MDS	Phase I	NCT02203903	/	
BAFF-R-CAR T Cells	BAFF-R	37	18 years and older	R/R B-cell ALL	Phase I	NCT04690595	/	

PD-1, programmed cell death-1; MRD, minimal residual disease; R/R, refractory/relapse; BCP ALL, B-cell precursor ALL; NHL, Non-Hodgkin Lymphoma; BAFFR, B-cell activating factor receptor; “/” represents that the detail information about the clinical information could be found in ClinicalTrials.gov or http://www.chictr.org.cn/.

## T Cell-Based Immunotherapy

**Figure 1:**
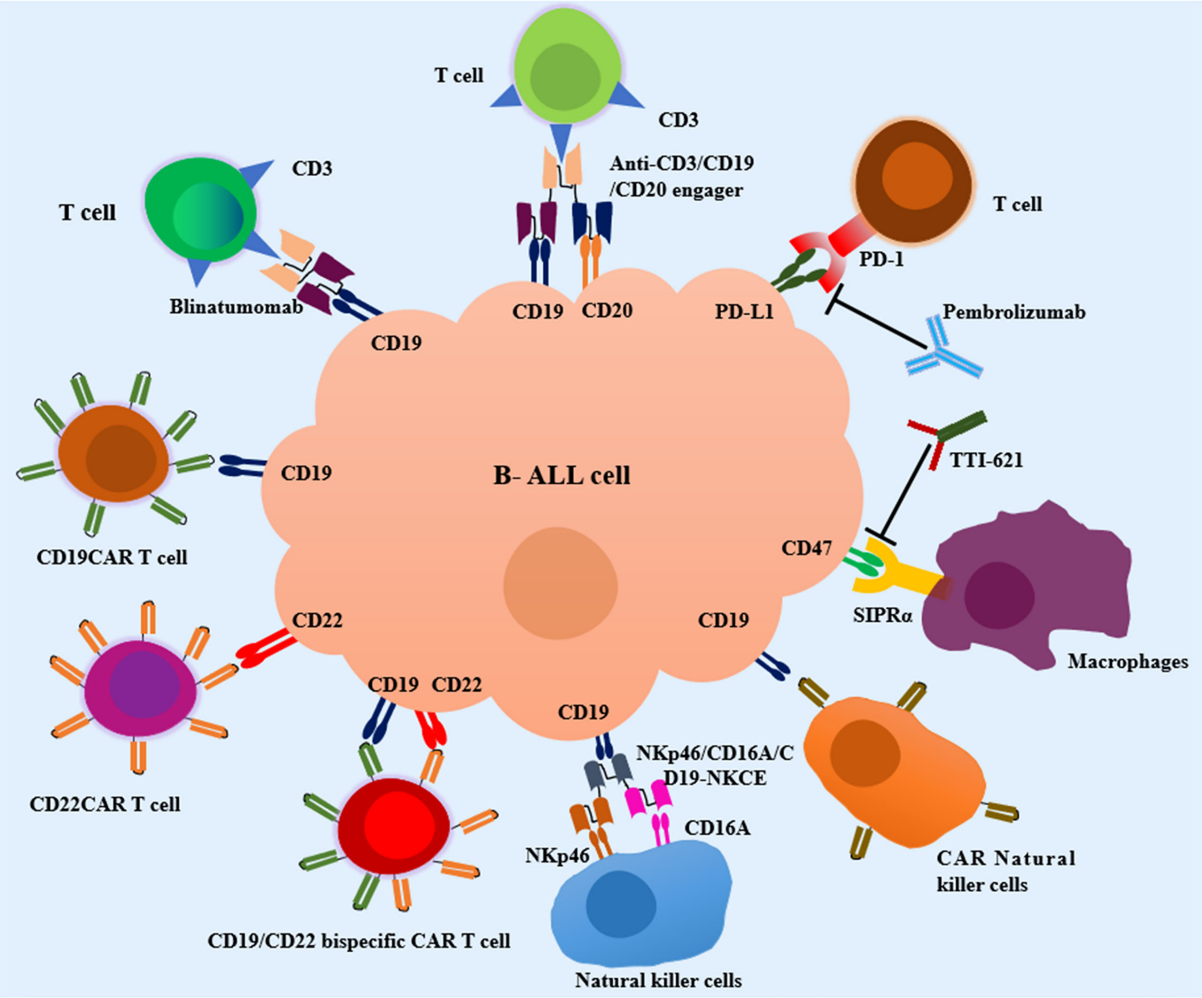
Current immunotherapeutic strategies in pediatric Acute Lymphoblastic Leukemia (ALL). T-cell engagers, CAR T cell therapy, macrophages-based immunotherapy, NK cell-based immunotherapy and other emerging immunotherapies are in development for pediatric B-cell ALL. SIRPα, signal regulatory protein alpha; NKp46/CD16A/CD19-NKCE, a NK cell engager with CD16A on the surface of NKs binding NKp46 and CD19 on the surface of B-cell ALL cells.

T cells have become an ideal weapon and attracted great research enthusiasm in cancer immunotherapy due to its capacity for antigen-directed cytotoxicity (45–47). Over the last decade, various T cell-based immunotherapeutic approaches, including blocking programmed cell death-1 (PD-1)/programmed cell death-ligand 1 (PD-L1) axis, bispecific/trispecific T-cell engagers, and chimeric antigen receptor (CAR) T cells have revolutionized the field of cancer therapeutics. The following context will highlight the T cell-based immunotherapeutic strategies available to attenuate pediatric B-cell ALL (**Figure 1**).

### Bispecific CD3/CD19 T Cell Engagers

Bispecific antibodies are designated to recognize and bind two distinct epitopes or antigens simultaneously, which have demonstrated great therapeutic potential toward cancer immunotherapy and are in rapid clinical development (48, 49). Blinatumomab, which is comprised of two different single-chain variable fragment regions (scFv) linked *via* a glycine-serine linker (50), triggers a cytotoxic immune response and shows significant cytotoxic activity at ultra-low concentrations, through binding specifically to antigen CD19 that is overexpressed on the surface of B-cell ALL lymphocytes and antigen CD3 on the surface of T cells (51–53). As the benchmarking case, blinatumomab conveyed good efficiency and safety in a phase I/II study (NCT01471782), which demonstrated that blinatumomab was maximumly tolerated at 15 μg/m^2^/day in 49 children with R/R BCP-ALL, 39% of whom achieved CR with single-agent blinatumomab treatment (24, 25). In the subsequent multi-center, expanded access study (RIALTO trial, NCT02187354), 63% of children had CR and the MRD negativity was obtained in 83% of responders after the first two cycles of blinatumomab treatment, further confirming the efficiency of blinatumomab (26, 27). Accordingly, blinatumomab was approved to treat R/R B-cell ALL in children by the US Food and Drug Administration (FDA) in 2018. Recently, two randomized trials (NCT02393859 and NCT02101853) exemplify the advantages of blinatumomab as post reinduction consolidation treatment vs chemotherapy before allogeneic hematopoietic stem cell transplant (alloHSCT), resulting in superior in eradicating MRD (28, 29). Therefore, blinatumomab gained accelerated approval by FDA to treat BCP ALL with MRD greater than or equal to 0.1% after the first or second CR. Moreover, a phase II study (NCT02807883) has proved the feasibility of blinatumomab maintenance following alloHSCT for patients with B-cell ALL at high-risk for relapse, with the 1-year overall survival (OS), progression-free survival (PFS), and nonrelapse mortality (NRM) rates of 85%, 71%, and 0%, respectively (30). In addition, to incorporate blinatumomab as part of upfront treatment for pediatric B-cell ALL, several trials are currently ongoing (NCT03643276 and NCT03914625) (31). Of note, the main toxicities including cytokine release syndrome (CRS) and neurotoxicity are tolerable under blinatumomab therapy (54, 55).

Although blinatumomab has obtained an authorization for treating pediatric R/R BCP ALL and eliminating the MRD, several disadvantages, such as the short half-life caused by the low molecular weight, leading to the need to continuous intravenous infusion, limited wide clinical application (56). Besides, blinatumomab resistance caused by the loss of CD19 expression and lineage switch in BCP ALL remains a significant problem (57, 58). For example, in a phase I/II study, four patients harboring CD19 negative B-cell ALL relapsed after prior blinatumomab-induced hematologic remission and one patient with CD19-negative had disease progression (57). In the future, screening tumor markers to predict CD19-negative relapse should be paid more attention. What’s more, overexpression of checkpoint molecules including T-cell immunoglobulin and mucin domain 3 (TIM-3) on T cells and PD-L1 on tumor cells represented an additional potential escape mechanism from immunosurveillance (30, 59, 60). Adding immune checkpoint inhibitors to blinatumomab treatment thus overcoming resistance may be feasible (60) and are under clinical investigation (NCT03160079, NCT03512405 and NCT04546399).

### Bispecific CD3/CD20 T-Cell Engagers

In addition to CD19, other antigens on the surface of leukemic blasts are currently under active research and development. CD20 is a signature B cell differentiation antigen and its overexpression is identified as an inferior prognosis marker associated with a worse event-free survival (EFS) in childhood BCP ALL according to the Pediatric Oncology Group treatment protocols (61). Also, CD20 expression was significantly up-regulated in pediatric B-cell leukemias during induction treatment (62), rendering it an appealing target for immunotherapy. Currently, several promising bispecific antibodies of anti-CD3/CD20, such as glofitamab, mosunetuzumab, REGN1979, and epcoritamab, may be of interest for future studies in B-cell Non-Hodgkin Lymphoma (NHL) (63, 64). However, further investigation is warranted to evaluate this strategy in pediatrics with B-cell ALL.

### Trispecific CD3/CD19/CD20 T-Cell Engagers

A-2019, a novel trispecific CD3/CD19/CD20 T-cell engagers possessing the anti-CD19 and anti-CD20 scFvs, was designed by Wang lab (65). It mediated autologous B cell depletion *ex vivo* by inducing the activation and proliferation of T cells, and the production and release of cytokines (65). Furthermore, A-2019 bound to CD19 with a lower affinity compared with blinatumomab (1.06 × 10^-8^ mol/L vs. 1.49 × 10^-9^ mol/L, respectively), which potentially reduce the off-target effect on mural cells, thus lowering the risk of neurotoxicity (66). Plus, a general decrease in overall cytokine including IL-6 and IFN-γ induced by A-2019 was observed in preclinical study, reducing the risk of CRS. In short, trispecific T-cell engagers targeting CD3/CD19/CD20 represents a novel strategy for not only preventing and treating CD19 negative relapse, but also has the potential for the treatment of CD20 positive and/or CD19 positive B-cell ALL. In a more recent preclinical study, CMG1A46, another CD3/CD19/CD20 T-cell engager generated from Chimagen’s TRIAD platform, displayed superior potency and safety in comparation with CD3/CD20 bispecific T-cell engagers (67). The phase I/II clinical trial is undergoing to evaluate the safety and efficacy of CMG1A46 in adult patients with advanced CD19 and/or CD20 positive B-cell NHL or ALL (NCT05348889).

### CAR T Cell Therapy

CAR T cells that commonly consists of an antigen-binding domain and costimulatory signaling domain such as CD28 and/or 4-1BB (68, 69), is a revolutionary and promising immunotherapy approach in cancer treatment (70). Antigen markers on B-cell ALL cells surface such as CD19 and CD22 can be specifically recognized by CAR that is independent from the major histocompatibility complex receptor, thus activating T cells to kill tumor cells.

#### CD19 Targeting CAR T Cell Therapy

Tisagenlecleucel, also named CTL019, is autologous T cells engineered *ex vivo* with a CAR containing a 4-1BB domain (71). In 2012, two children diagnosed with R/R B-cell ALL were infused with tisagenlecleucel and both achieved CR, although one patient had a CD19 negative relapse (72, 73). These encouraging data brought a hope of this therapy for the treatment of R/R B-cell ALL. Subsequently, a trial was expanded to 30 patients with the age of 5 to 22 years with R/R B-cell ALL in a phase I/IIa study (NCT01626495), which demonstrated a 90% rate of CR at the first month, an EFS rate of 67% and overall survival (OS) rate of 78% at 6 months after the single infusion of tisagenlecleucel (32). An international phase II study using tisagenlecleucel in pediatric and young adult patients with R/R B-cell ALL showed a CR rate of 81% at 3 months, EFS rate of 73%, and OS rate of 90% at 6 months (NCT02435849) (33). Given the unprecedented successes in clinical trials, tisagenlecleucel was commercially approved by the FDA and was indicated for R/R B-cell ALL patients up to 25 years old in 2017.

Brexucabtagene autoleucel, also named KTE-X19, is another CD19CAR T cell therapy with a CD28 costimulatory subunit generated from peripheral blood monocular cells by removing CD19 positive malignant cells to avoid T cell exhaustion, and has received FDA approval for mantle cell lymphoma (74). Based on the data from the pivotal phase I/II clinical trial (ZUMA-3, NCT02614066), brexucabtagene autoleucel was successfully manufactured and administered as a single infusion in 55 adult patients with R/R BCP ALL (34–36). Ultimately, 31 (56%) patients achieved CR and 8 (15%) patients achieved CR with incomplete haematological recovery, 38 (97%) of whom had MRD negativity. Due to the striking efficacy of brexucabtagene autoleucel in R/R BCP ALL adult patients, study to evaluate brexucabtagene autoleucel in pediatric and young adult patients with BCP ALL is ongoing (ZUMA-4, NCT02625480) and the results will be anticipated. Furthermore, several other CD19CAR T cell therapies are undergoing clinical research (**Table 1**) (75–77).

Although CD19CAR T cell therapy has shown great success for pediatric B-cell ALL, some patients displayed no response and a great part of patients suffered from relapse with poor outcomes (NCT01593696) (37, 38). Unfortunately, even a secondary infusion of CD19CAR T cells can’t prevent CD 19-positive relapse, which may be partly due to immune-mediated clearance of CAR T cells (78, 79). Antigen loss caused by mutations or alternate splice variants of CD19 have been elucidated as the major resistance mechanism to CD19CAR T cell immunotherapy, which is widely acknowledged as an urgent problem to be solved (80, 81). Therefore, new therapeutic approaches are required for R/R B-cell ALL patients who have failed previous CD19CAR T cell therapy.

#### CD22 Targeting CAR T Cell Therapy

CD22 represents an alternative target with a high expression level on most B-cell ALL cells, while has restricted expression on normal B cells, especially in the absence of CD19 expression (82–84). Recently, a phase I dose escalation study of CD22CAR T cells with a 4-1BB costimulatory domain in pediatric and young adults with recurrent or refractory CD22 positive B cell malignancies was conducted sponsored by National Cancer Institute (NCT02315612). The results showed that 70.2% of the patients achieved CR and 87.5% of whom were MRD negative (39, 85). However, an CD22CAR possessing the similar structure with the above mentioned CD22CAR but the heavy and light chains were connected by a standard 20-aminoacid linker instead of a short 5-amino acid sequence, proved surprisingly poor response in pediatric and adult patients with B-cell ALL (NCT02588456) (40). By performing detailed interrogation responsible for the entirely different findings from the two independent clinical trials, mechanisms that short scFv linker and tonic signaling enhanced the antileukemic function of 4-1BB-based CAR T cells induced the phenomena (40). Based on this work, the pilot study (NCT02650414) was amended to determine the feasibility and safety of a single dose administered CD22CAR T cells expressing 4-1BB costimulatory domains in pediatric R/R B-cell ALL patients (40). Moreover, Pan and his colleges constructed an CD22CAR with a 4-1BB costimulatory domain and initiated a clinical trial, which demonstrated a CR rate of 80% on day 30 after infusion in 34 R/R B-cell ALL pediatric and adult patients who have mostly failed from first CD19CAR T cell therapy (ChiCTR-OIC-17013523) (41). However, most patients relapsed with a diminished CD22 site density on B-cell ALL cells (85), which raised the question that single target therapy permit the occurrence of resistant variants. Combinatory or tandem CARs, which contain two or more antigen-recognition moieties, may prevent from relapse due to escape variants but need further validation (86, 87).

#### Dual CD19 and CD22 Targeting CAR T Cell Therapy

The downregulation or loss of pre-designed antigen on ALL cells leads to the failure of CD19CAR T or CD22CAR T cell monotherapy. Therefore, a dual CD19 and CD22 antigen targeting CAR T cell therapy such as CD19/CD22 bispecific CAR T cell therapy and sequential CD19CAR T and CD19CAR T cell therapy, appears to be a strategy to prevent the escape mechanism given that B cells are unlikely to downregulate both CD19 and CD22 simultaneously in a single cell.

AUTO3 is a representative CD19/CD22 bispecific CAR T cell therapy, which is designed and developed through transduction of autologous T cells expressing two CARs targeting CD19 and CD22 by Autolus Therapeutics. A multi-center, phase I/II study has been completed to explore the safety and efficacy in pediatric and young adult patients with B-cell ALL (AMELIA trial, NCT03289455) (42). In the phase I dose-escalation study, dose-limiting toxicities, severe CRS, and neurotoxicity were not reported, which demonstrated a favorable safety profile for clinical application. 13 of 15 patients achieved CR or CR with incomplete bone marrow recovery after AUTO3 infusion for one month. The OS and EFS rates were 60% and 32%, respectively. Consequently, FDA has granted orphan drug designation of AUTO3 for ALL treatment. Nevertheless, unavailability of long-term persistence of AUTO3 in patients resulted in disease relapse. Hence, prolongation of CAR T cell persistence are needed to fully fulfill the therapeutic potential of dual targeting CAR T cell in B-cell ALL. Another phase I trial (NCT03448393) sponsored by National Cancer Institute to evaluate the safety and efficiency of dual CD19/CD22 targeting CAR T cell therapy is undergoing.

CTA101, also called CRISPR-edited allogeneic off-the-shelf CD19/CD22 bispecific CAR T cells, was composed of scFV targeting CD19 and CD22, 4-1BB costimulatory domain, and CRISPR/Cas9-disrupted *TRAC* region to avoid host immune-mediated rejection (88). The phase I clinical trial (NCT04227015) to evaluate the safety and efficiency of a single dose of CTA101 in R/R B-cell ALL patients aged from 3 to 70 years is ongoing.

In addition to CD19/CD22 bispecific CAR T cells, sequential infusion of CD19CAR T and CD22CAR T cells are investigated in a phase I trial in pediatric patients with R/R B-cell ALL (ChiCTR-OIB-17013670). 17 of the 20 patients remained CR at the study end point and only 2 patients relapsed caused by loss of CD19, indicating that the risk of relapse associated with antigen escape was greatly reduced (43).

#### Strategies to Reduce Toxicities of CAR T Cell Therapy

Despite the huge clinical success, CAR T cell therapy-related severe toxicities such as CRS, cannot be neglected and are remained to be resolved (33, 89, 90). For example, around 50% of children and young adult patients treated with tisagenlecleucel for R/R B-cell ALL had ≥ Grade 3 CRS (33, 91), 24% of adult patients infused with brexucabtagene autoleucel had ≥ Grade 3 CRS (36), while lower incidence of ≥ Grade 3 CRS, 8.6% and 2.9% respectively, was observed after CD22-CART cell treatment (39, 41). Moreover, no ≥ Grade 3 CRS was observed in pediatric patients with R/R B-cell ALL receiving AUTO3, which is in concordance with the modest elevation of cytokines production (42). Finally, extensive studies to ameliorate CAR T cell related toxicities are ongoing, such as altering CAR structure, modifying CAR transduced T cells, and inserting CAR “off-switches” (70, 92, 93). Optimizing CAR binding affinity by developing a new CD19 scFV with a lower affinity than FMC63 could be a useful approach to enhance CAR T cell expansion and persistence, and alleviate toxicity in the pediatric R/R B-cell ALL as illustrated in the CARPALL clinical trial (NCT02443831) (44).

#### Strategies to Increase the Efficacy of CAR T Cell Therapy

Besides reducing toxicities of CAR T cell therapy, attempts have been made to increase the efficacy of CAR T cell therapy. As mentioned above, targeting two or more antigens by dual CD19 and CD22 targeting CAR T cells or sequential infusion of CD19CAR T and CD22CAR T cells broadened the spectrum of targets, decreased the risk of antigen negative relapse and enhanced the potential therapeutic efficiency. Moreover, tisagenlecleucel and other CAR T cell therapies for pediatric B-cell ALL were commonly composed of the costimulatory domains with CD28 and/or 4-1BB (**Table 1**), which were the second and third generation CARs (94). CAR T cells were further optimized to secrete cytokines or express cytokine receptors to generate the fourth and fifth generation CARs. In a relapsed patient with B-cell ALL after treatment with CD19CAR and CD22CAR T cell therapy, autologous murine CD19CAR T cells expressing membrane-bound IL-15 achieved CR for five months (95). CD19CAR T cells encoded with interleukin 2 receptor β-chain and a STAT3-binding tyrosine-X-X-glutamine motif in the cytoplasmic domain showed grater antitumor effects and superior duration than CAR T cells without this structure (96). Besides, upregulation of TIM-3 increased the risk of relapse in pediatric B-cell ALL (97). Hence, TIM-3-CD28 fusion proteins were combined with the first and second CD19CAR T cells to enhance the proliferation capacity of T cells and improve the functionality of conventional CAR T cells by turning inhibition into activation of T cells (98, 99). Relapse due to the short persistence of CAR T cells and resistance to same murine CAR T cell therapy were attributed to immunogenicity caused by murine scFv (37, 38). 68% of the pediatric and adult patients after failure of murine CD19CAR T cell therapy achieved CR treated with 4-1BB based humanized CD19 CAR-T cells (ChiCT R1900 024456) (100). In brief, various strategies have been developed to increase the efficacy of CAR T cell therapy and warranted to be applied in the treatment of pediatric B-cell ALL.

### Combination Therapy

Numerous studies have demonstrated that immune responses by maintaining negative regulatory pathways *via* PD-1/PD-L1 axis played a significant role in the immune escape, thus leading to growth and spread of malignant tumors (101, 102). In this regard, blocking PD-1/PD-L1 pathway has aroused great success as an effective therapeutic approach in a variety of cancers. Significantly, there is a high expression level of PD-1 on the surface of T cells in B-cell ALL pediatric patients, which is associated with an inferior prognosis (103). Inspired by the encouraging therapeutic outcomes of blocking the PD-1/PD-L1 pathway in the treatment of solid tumors, researchers nowadays have tried to integrate PD-1/PD-L1 inhibitors into the treatment course of hematological malignancy (104, 105). Pembrolizumab is a humanized anti-PD-1 monoclonal antibody with a wide range of indications (106). However, the phase II study of single-agent pembrolizumab in treating minimal residual disease (MRD) in adults with ALL was terminated due to lack of efficacy despite good tolerability (NCT02767934) (107). Immunosuppression due to chemotherapy prior treatment and relatively low mutational rate may lead to the treatment failure of targeting PD-1/PD-L1 pathway (107). That said, monotherapy by blocking PD-1/PD-L1 signaling pathway seems not a viable therapeutic method for the treatment of ALL. Nevertheless, combinatory treatment of immune checkpoint inhibitors including pembrolizumab and nivolumab with blinatumomab for B-cell ALL in adults and pediatrics are undergoing clinical trials (NCT03160079 and NCT03512405 and NCT04546399), which may prove the potential advantage of anti PD-1 antibody as an adjunct or rescue strategy. Besides immune checkpoint inhibitors, blinatumomab in combination of with chemotherapy in patients with newly diagnosed B-lymphoblastic leukemia aged 365 days to 31 years are being investigated (NCT03914625).

### Other Emerging T-Cell Based Immunotherapies

Donor-derived tumor associated antigen-specific T cells termed TAA-T (108), reported by Kinoshita and colleges, is capable of targeting three overexpressed and immunogenic tumor associated antigens WT1, PRAME and survivn in most hematologic malignancies (109–111). A phase I clinical is currently ongoing at Children’s National and Johns Hopkins Hospitals and Johns Hopkins University to evaluate the safety of TAA-T for the treatment of very high-risk hematopoietic malignancies (NCT02203903). Preliminary results showed that none of the included acute myeloid leukemia (AML) and ALL patients developed CRS or neurotoxicity, and only one patient developed grade 3 graft-versus-host disease (GVHD), which demonstrated the good safety of TAA-T (108). Moreover, persist remissions were observed in high-risk and relapsed patients (108). Later phase of the study is required to determine long-term clinical disease outcomes.

Adoptive cell therapy using T-cell receptor (TCR)-engineered T cells represents another novel and potential strategy for cancer treatment (112–114). ET190L1-AbTCR is one of TCR-T cell therapies generated by replacing the α and β chains of the TCR in the antigen recognition domain with an anti-CD19 antibody-derived Fab fragment (115). Compared with the clinically commonly used CD19CAR T cells with a CD28 or 4-1BB costimulatory subunit, ET190L1-AbTCR activated cytotoxic T-cell responses, but showed less cytokine release in xenograft mouse models of primary B-cell ALL.

B-cell activating factor receptor (BAFF-R) is a B-lineage marker expressed almost exclusively on B cells (116, 117), making it an ideal target for immunotherapy. BAFF-R-CAR T cells with a 4-1BB costimulatory signaling domain demonstrated therapeutic effects against CD19 negative B-cell ALL *in vitro* and *in vivo* (117), and are currently undergoing clinical trials for the treatment of adult ALL (NCT04690595). Moreover, dual CD19/BAFF-R CAR T cells were developed and exhibited anti-ALL activity *in vivo*, supporting clinical translation of BAFF-R/CD19 dual CAR T cells to treat ALL (118).

## Macrophage-Based Immunotherapy

### Targeting CD47/SIRPα Pathway

Macrophages are important components of mononuclear phagocytic system, and generates a “don’t eat me” signal to suppresses phagocytosis by expressing a signal regulatory protein alpha (SIRPα) that interacts with CD47 (CD47/SIRPα axis), thus contributing to the development and progression of most cancers (119–121). Therefore, the CD47/SIRPα axis has been identified as an essential and promising immune checkpoint in the homeostatic clearance by macrophages. Generally, CD47 is overexpressed on the surface of B-cell ALL cells and recognized as an inferior prognosis marker associated with worse outcomes in pediatric ALL patients, such as treatment failure and even death (122, 123). Additionally, the anti-CD47 antibody and anti-SIRPα antibody both increased phagocytosis of ALL cells *in vitro*, suggesting that blockade of the CD47/SIRPα signaling enhanced phagocytosis (123). *In vivo*, anti-CD47 antibody inhibited tumor engraftment and induced remission in ALL engrafted mice (123). These data provided pre-clinical evidence for disrupting CD47/SIRPα signaling as a potential therapy for ALL.

TTI-621 is a novel soluble fusion protein composed of human SIRPα and IgG1, and exerts its effect by blocking CD47/SIRPα pathway (124) (**Figure 1**). Recently, a phase Ia/Ib dose escalation and expansion trial of TTI-621 in R/R hematologic malignancies and selected solid tumors is ongoing (NCT02663518). TTI-621 was well-tolerated in the dose escalation phase. The expansion phase conducted in R/R NHL patients has been completed and demonstrated a sound efficacy. The evaluation of clinical efficacy and safety of TTI-621 in ALL patients is still ongoing.

Additionally, several strategies have been proposed and are being intensively explored, for example, anti-CD47 antibody (124–128), anti-SIRPα antibody (129), bi-specific antibodies to CD47 or SIRPα or other molecules (129), SIRPα-related fusion proteins (130), and others (131), to improve therapeutic efficacy during targeting CD47/SIRPα pathway while overcome on-target/off-tumor effects (132, 133). A large number of clinical trials related to cutting off the CD47/SIRPα pathway are currently undergoing at various stages, which are centered on solid tumors and hematological malignancies such as NHL and AML (132). The research on blocking the CD47/SIRPα pathway in pediatric ALL is lagging behind and is undergoing preclinical study.

Notably, targeting CD47 induced hematotoxicity including anemia and thrombocytopenia due to the off-target effect on the blocking of CD47 expressed on surface of platelets and erythrocytes (134). Designing and developing SIRPα fusion proteins could diminish the hematotoxicity, thereby safeguarding the clinical safety. For example, TTI-621 exhibited minimal impact on human erythrocytes, resulting in a lower incidence of anemia than that reported in a phase I study of combinatory treatment of humanized anti-CD47 antibody and rituximab (13% *vs* 41%) (124, 135). Moreover, thrombocytopenia associated with TTI-621 was transient and reversible, and no bleeding events were observed in the clinical trials (136).

## NK Cell-Based Immunotherapy

NKs derived from lymphocyte cell lineage were discovered to be critical to the innate immunity (137). NKs are composed of two subtypes, i.e., CD3^-^/CD56^dim^/CD16^+^ NKs and CD3^-^/CD56^bright^/CD16^-^ NKs (138). CD3^-^/CD56^dim^/CD16^+^ NKs display strong cytotoxic activity on targeted cells by perforin and granzyme B in peripheral blood, and CD3^-^/CD56^bright^/CD16^-^ NKs produce and release cytokines such as IFN-γ and TNF-α in response cytokines stimulation in lymphoid tissues (139, 140). Mounting studies have demonstrated the innate lymphoid cells as the first line of defense by exerting cytotoxicity against diverse tumor cell types (141, 142), especially in the field of hematologic malignancies (143). The presence of NK cells in bone marrow conferred a better response to chemotherapy and prognosis, and has a high chance of efficacy in pediatric patients with ALL (144, 145). Hence, different artificial engagers have been equipped to selectively redirect NK cells towards tumor cells. *In vitro*, NKp46/CD16A/CD19-NKCE, a NK cell engager with CD16A on the surface of NKs binding NKp46 and CD19 highly expressed on the surface of B-cell ALL cells, enhanced the activation of NKs and promoted NK cell-mediated lytic effects against pediatric BCP ALL (**Figure 1**) (146).

Moreover, immunotherapy based on NKs displayed several advantages (147, 148). As the important immune system components, NKs exerted the cytotoxicity upon recognizing specific patterns without prior antigen sensitization. CAR modified NK cells (CAR NKs) are qualified for simultaneously improving efficacy and controlling adverse effects including CRS, neurotoxicity, and GVHD, which offer an alternative option to CAR-T cells (149, 150). To date, two phase I studies to determine the maximum tolerated dose of genetically modified haploidentical NKs infusions targeting CD19 to treat B-cell ALL have been completed in St. Jude Children’s Research Hospital (NCT00995137 and NCT01974479). However, the results have not been disclosed.

## Conclusion and Future Perspectives

Immunotherapy represents a novel and promising therapeutic weapon against pediatric B-cell ALL. As alluded to above, T cell engagers and CAR T cell therapies were successful in treating pediatric R/R B-cell ALL. Currently, blinatumomab has been approved for treating R/R BCP ALL in pediatrics and is further investigated in removing MRD as post-reinduction consolidation treatment, which is believed to be more efficacious and less toxic than chemotherapy. In addition, the approval of tisagenlecleucel, a typical representative of CAR T cell therapies, provided us an alteration when none was available for children and young adults with R/R B-cell ALL, although a large proportion of patients suffered relapse with poor outcomes. However, monotherapy by immune checkpoint inhibitors targeting PD-1/PD-L1 pathway achieved poor clinical efficiency. Immune check inhibitors including anti-PD-1 and anti-CD47 antibodies as an adjunct strategy in pediatric B-cell ALL required to be verified in the further study and may be worth awaiting. Moreover, strategies targeting CD47/SIRPα pathway and CAR-NKs demonstrated potential for treating pediatric ALL and were evaluated in clinical trials.

Since native macrophages are critical effectors and regulators of the innate immune system, CAR macrophages can directly target the desired cancer cells and has been developed to phagocytose human solid tumor cells (151, 152). Nevertheless, treatment of pediatric ALL by using this promising immunotherapy is absent (153). Neoantigen, a new mutation-derived protein in tumor cells, is a non-normal cellular product and has proven to be a breakthrough for therapeutic immune targets (154–157). Immunotherapy by harnessing neoepitope-CD8+ T cells to recognize and respond to the neoantigens in pediatric patients with ALL provided us an alternative treatment option (158).

Despite all these advances, the resistance and toxicity should be taken into consideration and further studies should focus on the development of new agents guided to ameliorate some of the toxicities and prevent the recurrence. Variable mechanisms including the downregulation/loss of antigens and antigen escape through lineage switch influence responses to immunotherapy result in resistance or relapse. The relationship between genetic and cytogenetic alterations and immunotherapy responses are warranted to be further explored in the future. Moreover, novel potential targets such as cell surface antigens, kinases and signaling pathways should be consistently identified and explored. On the other hand, blocking a single cell surface antigen or pathway of B-cell ALL may lead to drug resistance. It is anticipated that immunotherapy targeting multiple antigens such as trispecific CD3/CD19/CD20 T-cell engagers and dual CD19 and CD22 targeting CAR T cell therapy will not only overcome the challenge of antigen modulation but also dramatically enhance therapeutic efficacy.

To sum up, the successful outcome of immunotherapy from clinical trials as described in our context has proved the effectiveness of immunotherapy in the treatment of pediatric B-cell ALL and lead to dramatic improvements in outcome for R/R subtype. We believe that the integration and expansion of these therapeutics into frontline therapy and the discovery of novel anti-multiple antigens modifications will further augment efficacy and reduce toxicity, thus improving long-term outcomes in pediatric B-cell ALL patients.

## Author Contributions

ML and YL designed the study, drafted the initial manuscript, critically reviewed and revised the manuscript, and contributed equally to this work. WL provided us professional knowledge in the field of pediatric ALL. YX and SZ reviewed and revised the manuscript. All authors contributed to the article and approved the submitted version.

## Conflict of Interest

The authors declare that the research was conducted in the absence of any commercial or financial relationships that could be construed as a potential conflict of interest.

## Publisher’s Note

All claims expressed in this article are solely those of the authors and do not necessarily represent those of their affiliated organizations, or those of the publisher, the editors and the reviewers. Any product that may be evaluated in this article, or claim that may be made by its manufacturer, is not guaranteed or endorsed by the publisher.
